# Attention-Deficit/Hyperactivity Disorder Mimics the Post-concussion Syndrome in Adolescents

**DOI:** 10.3389/fped.2020.00002

**Published:** 2020-02-05

**Authors:** Nathan E. Cook, Rosemarie G. Sapigao, Noah D. Silverberg, Bruce A. Maxwell, Ross Zafonte, Paul D. Berkner, Grant L. Iverson

**Affiliations:** ^1^Department of Physical Medicine and Rehabilitation, Harvard Medical School, Boston, MA, United States; ^2^Sports Concussion Program, MassGeneral Hospital for Children, Boston, MA, United States; ^3^Spaulding Rehabilitation Hospital, Charlestown, MA, United States; ^4^Massachusetts General Hospital, Boston, MA, United States; ^5^T.H. Chan School of Public Health, Harvard University, Boston, MA, United States; ^6^Division of Physical Medicine and Rehabilitation, University of British Columbia, Vancouver, BC, Canada; ^7^Rehabilitation Research Program, Vancouver Coastal Health Research Institute, Vancouver, BC, Canada; ^8^Department of Computer Science, Colby College, Waterville, ME, United States; ^9^Brigham and Women's Hospital, Boston, MA, United States; ^10^Health Services and Department of Biology, Colby College, Waterville, ME, United States; ^11^Spaulding Research Institute, Boston, MA, United States

**Keywords:** concussion, mild traumatic brain injury, pediatrics, assessment, symptoms

## Abstract

The objective of this study was to evaluate concussion-like symptom reporting among uninjured adolescents with Attention-deficit/hyperactivity disorder (ADHD), stratified by several cooccurring conditions, and to examine the base rate and predictors of uninjured adolescents with ADHD meeting diagnostic criteria for the International Classification of Diseases, 10th Revision (ICD-10) post-concussional syndrome (PCS). Participants in this cross-sectional, observational study, were drawn from a cohort of 48,834 adolescent student athletes from Maine (ages 13–18) with no concussion in the past 6 months who completed a preseason, baseline testing program between 2009 and 2015. The final sample included 3,031 students with ADHD, 2,146 (70.8%) boys and 885 (29.2%) girls. They were 15.2 years old on average (SD = 1.3). Concussion-like symptom reporting was more common in girls than boys. Most students with ADHD reported one or more symptoms (69.3% of boys and 81.1% of girls). The presence of an additional, co-occurring condition or comorbidity was associated with increased symptom reporting. In the absence of a recent concussion, 28.8% percent of boys and 47.1% of girls with ADHD endorsed symptoms resembling an ICD-10 diagnosis of post-concussional syndrome (PCS). Adolescents with pre-existing conditions were even more likely to endorse symptoms that resembled PCS (28–47% of boys and 45–69% of girls). Prior treatment for a psychiatric condition was the strongest independent predictor for meeting PCS criteria in boys, followed by treatment for migraines and co-occurring learning disorder. For girls, the only independent predictor was prior treatment of a psychiatric condition. In uninjured adolescent student athletes, ADHD appears to mimic the post-concussion syndrome. Adolescents with ADHD commonly endorse concussion-like symptoms in the absence of a recent concussion. Demographic characteristics (sex) and the presence of co-occurring conditions are related to symptom reporting in adolescents with ADHD. Understanding factors associated with baseline symptom reporting, such as pre-existing ADHD, is important when evaluating youth who have persistent symptoms following concussion as well as making both return to school and return to athletics decisions.

## Introduction

Attention-deficit/hyperactivity disorder (ADHD) is a common neurodevelopmental disorder, occurring in roughly 10% of children and adolescents (1), or more than 5 million youth in the United States (2). Children and adolescents with ADHD often seek care through their primary care physician (PCP)/pediatrician. In fact, pediatricians are the most common health care provider to make a first diagnosis of ADHD (3) and are the sole treatment provider for about 42% of youth with ADHD (4). Participation in sports and engaging in physical exercise can have positive effects on youth with ADHD. For example, children with ADHD who participate in a greater number of sports exhibit lower levels of anxiety and depressive symptoms compared to children with ADHD who participate in fewer or no sports (5). Also, even a single, brief period of aerobic exercise can lead to improved inhibitory control and reaction time in children with ADHD (6). Participation in sports, however, carries an inherent risk for injury, including concussion.

It has been estimated that 33 million children worldwide sustain a concussion annually (7). In the United States, an estimated 1.1–1.9 million sports- and recreation-related concussions occur each year (8). Pediatrician offices represent the most common initial point of care following concussions, as 82% of patients sought their initial concussion care within a primary care setting compared to 12% who presented initially to an emergency department or urgent care setting (9). Moreover, a study of trends in ambulatory care for children with concussion noted a nearly 500% increase in PCP visits for concussion from 2007 to 2013, compared to a 42% increase in emergency department visits for this injury (10). In a recent survey of pediatricians, half (50%) reported treating six or more concussed patients over the past year and essentially all of them (99%) reported treating at least one concussed patient over that time (11).

Physicians and other health care providers use symptom questionnaires as part of their diagnostic evaluation, to monitor clinical recovery, and to inform return to activity decision making (12). A challenge many providers face is that concussion symptoms are non-specific, which complicates the interpretation of symptom questionnaires—especially when a youth has prolonged symptoms and other health conditions or life stressors that can be contributing to the symptoms. This appears especially true for pediatric patients with ADHD. Youth with ADHD endorse many concussion-like symptoms in their daily lives, in the absence of recent head trauma (13–15). In a large study examining symptom reporting among uninjured adolescent athletes, those with ADHD endorsed considerably more physical, emotional, and cognitive symptoms than those without ADHD (14). In fact, nearly one-third of uninjured boys (31.5%) and one-half of uninjured girls (52.6%) with ADHD endorsed symptoms across a number of domains of functioning and would meet International Classification of Diseases, 10th Revision (ICD-10) symptom criteria for post-concussional syndrome (PCS) (14), a condition marked by a variety of physical/somatic, cognitive, sleep, and emotional symptoms associated with a brain injury (16). Also of note, the presence of comorbid learning disorder increased the likelihood that youth with ADHD met criteria for PCS in the absence of recent injury (14). Thus, the presence of co-occurring conditions is highly relevant when considering symptom reporting among youth with ADHD.

For clinicians treating adolescents with ADHD who sustain a concussion, understanding factors associated with baseline symptom reporting is important when determining recovery from the injury as well as making decisions regarding return to school and return to sports. The goal of this study was to examine symptom reporting among uninjured adolescents with ADHD using a statewide dataset of over 48,000 student athletes. We sought to extend prior work in this area by stratifying symptom reporting among youth with ADHD by the presence or absence of several potentially important co-occurring health conditions. We hypothesized that many adolescents with ADHD would report concussion-like symptoms in the absence of recent injury, and that demographic factors (e.g., female sex) and co-occurring health conditions (e.g., learning disorders, history of treatment of psychiatric conditions, history of treatment for migraines, and multiple prior concussions) would be associated with greater baseline symptom reporting.

## Methods

### Participants

Participants in this multi-year, cross-sectional, descriptive study were drawn from a cohort of 48,834 adolescent student athletes from Maine, USA, between the ages of 13 and 18 (*M* = 15.0, SD = 1.3 years), who completed baseline preseason testing with the Immediate Post-Concussion Assessment and Cognitive Testing (ImPACT®) (17) between 2009 and 2015. There were 26,736 (54.7%) boys and 22,098 girls (45.3%). Students were excluded if (i) they reported sustaining a concussion within the past 6 months (*n* = 1,207); (ii) they reported a history of treatment for epilepsy/seizures (*n* = 495) or meningitis (*n* = 201), or reported undergoing brain surgery (*n* = 102); or (iii) they completed the test in a language other than English (*n* = 814). Of the remaining 46,125 eligible adolescents (94.4% of the original sample), 3,031 self-reported having ADHD (6.6% of the eligible sample) and represent the final sample for this study. The adolescents with ADHD included 2,146 (70.8%) boys and 885 (29.2%) girls. They were 15.2 years old on average (SD = 1.3). Adolescents completed ImPACT® prior to participating in their first sport for that school year. Institutional review board approval to create and use this de-identified database was obtained from Colby College (primary) and from Spaulding Rehabilitation Hospital (secondary use).

### Measures

A demographics and health history survey is embedded in ImPACT, a computerized neuropsychological screening battery designed for use in sport concussion programs (17). The health history survey asked the student whether he or she has “had problems with ADD/hyperactivity,” been diagnosed with a learning disability, received special education services, or repeated a grade. These questions require a yes or no response. The health history survey also asks about the number of times the student has been diagnosed with a concussion as well as about past treatment for headaches, migraines, epilepsy, brain surgery, meningitis, substance use, or a psychiatric condition (e.g., anxiety or depression). Athletes can enter the names of medications they are taking in an open text field in the health survey; self-reported medication status was not analyzed in this study.

The Post-Concussion Symptom Scale (18, 19) is a component of ImPACT. This standardized self-report questionnaire includes 22 symptoms (see Tables 1, 2) that are rated from zero to six in terms of severity, with one or two reflecting “mild,” three or four reflecting “moderate,” and five or six reflecting “severe” problems with a given symptom. The internal consistency of the scale ranges from 0.88 to 0.94 in high school and college students, and 0.92 to 0.93 in concussed athletes (19). Students completed the Post-Concussion Symptom Scale independently.

**Table 1:** Percentages of boys with ADHD endorsing individual symptoms on the Post-Concussion Scale, stratified by pre-existing health condition and concussion history.

								**Concussion history**			
	**All boys** ** with** ** ADHD**	**No other** ** pre-existing** ** condition**	**Learning** ** disability**	**Academic** ** problems**	**Migraine**	**Psych.** ** Tx Hx**	**Substance** ** Tx Hx**	**None**	**1**	**2**	**3+**
	***n* = 2,146**	***n* = 709**	***n* = 408**	***n* = 635**	***n* = 190**	***n* = 414**	***n* = 70**	***n* = 1,601**	***n* = 315**	***n* = 119**	***n* = 74**
Headache^a^	18.3	13.5^**^	18.6	18.9	34.7^**^	23.7^**^	**21.4**	17.2	18.1	25.2^*^	31.1^**^
Vomiting	3.7	2.1^*^	4.2	5.7^**^	7.4^**^	4.8	2.9	3.9	1.9	4.2	6.8
Nausea^a^	4.3	2.1^**^	6.6^*^	6.5^**^	9.5^**^	7.2^**^	8.6	4.4	2.2	5.9	9.5
Balance problems^a^	6.3	2.1^**^	7.1	9.1^**^	10.5^*^	10.1^**^	12.9^*^	5.7	6.3	10.1	13.5^*^
Dizziness^a^	8.6	5.4^**^	11.0	10.9^*^	14.7^**^	11.4^*^	7.1	8.5	7.3	10.1	13.5
Trouble falling asleep^d^	**28.0**	22.7^**^	**30.4**	**29.6**	**33.7**	42.3^**^	45.7^**^	**27.9**	**27**	**28.6**	**32.4**
Fatigue^a^	**22.5**	17.8^**^	**25.7**	**21.3**	29.5^*^	30.4^**^	**31.4**	**21**	**25.4**	**21.0**	39.2^**^
Sleeping more than usual	10.5	6.9^**^	12	11.8	10.5	14.3^**^	10.0	10.2	13.3	9.2	5.4
Sleeping less than usual^d^	**25.7**	22.1^**^	31.1^**^	**27.9**	**30.0**	35.0^**^	38.6^*^	**24.9**	**26**	**31.1**	**32.4**
Sensitivity to light^a^	12.4	8.9^**^	14.2	13.9	20.5^**^	15.2^*^	12.9	11.1	13.7	14.3	21.6^*^
Drowsiness	18.6	14.1^**^	**20.1**	**20.0**	26.8^**^	32.4^**^	35.7^**^	18.1	19.0	16.0	36.5^**^
Sensitivity to noise^a^	7.8	4.5^**^	8.8	9.8^*^	15.3^**^	12.8^**^	10.0	7.1	9.8	8.4	10.8
Irritability^b^	17.9	11.3^**^	22.1^*^	21.9^**^	23.7^*^	33.8^**^	34.3^**^	17.5	17.8	**22.7**	**21.6**
Nervousness^b^	19.1	11.3^**^	28.4^**^	24.9^**^	28.4^**^	36.7^**^	**28.6**	17.9	**22.2**	**24.4**	**21.6**
Sadness^b^	15.7	8.9^**^	24.0^**^	22.0^**^	**20.0**	31.6^**^	31.4^**^	14.9	16.8	17.6	17.6
Feeling more emotional^b^	13.7	7.6^**^	18.9^**^	16.9^**^	21.1^**^	27.8^**^	**20.0**	12.9	14.6	19.3	14.9
Numbness or tingling	5.1	3.4^**^	7.1	7.4^**^	8.9^*^	8.0^**^	4.3	5.0	5.4	6.7	4.1
Feeling mentally “Foggy”^c^	10.9	6.3^**^	13.7^*^	13.7^**^	13.2	19.6^**^	30.0^**^	10.1	11.4	13.4	14.9
Feeling slowed down	11.4	7.1^**^	15.0^*^	13.9^*^	17.9^**^	18.4^**^	15.7	10.9	11.4	12.6	16.2
Difficulty concentrating^c^	**38.4**	31.0^**^	43.6^*^	40.8^*^	44.7^*^	50.0^**^	55.7^**^	**38.5**	**34.0**	**46.2**	**40.5**
Difficulty remembering^c^	16.6	11.7^**^	23.3^**^	19.1^*^	18.4	25.1^**^	30.0^**^	16.8	13.3	17.6	**25.7**
Visual problems	9.0	6.2^**^	9.8	11.5	15.3^**^	15.0^**^	7.1	8.9	7.6	10.1	16.2
No symptoms	**30.7**	37.9^**^	**27.2**	**28.5**	**25.3**	15.2^**^	18.6^*^	**31.4**	**29.2**	**24.4**	**27.0**
**Post-concussion scale**											
Mean	7.3	4.6	9.9	8.9	11.5	12.7	12.2	6.9	7.2	8.9	11.9
Median	3.0	2.0	6.0	4.0	8.0	8.0	8.5	3.0	4.0	5.0	7.0
SD	10.6	6.9	12.6	12.4	13.1	14.2	12.1	10.3	10.0	11.1	14.2
25th percentile	0.0	0.0	0.0	0.0	0.0	3.0	3.0	0.0	0.0	1.0	0.0
75th percentile	10.0	6.0	14.0	12.0	17.0	19.0	17.3	9.0	10.0	13.0	16.5
90th percentile	20.0	14.0	27.0	26.0	30.9	30.0	28.9	19.0	19.0	26.0	33.5

*All values are percentages endorsing the symptom as mild or greater. Symptoms endorsed by 20% or more of each group are bolded*.

*ADHD, Attention Deficit Hyperactivity Disorder; Tx, Treatment; Hx, History*.

*Symptoms counted toward each criterion for ICD-10 Post-concussional Syndrome*:

a *Category 1 (physical) symptoms*,

b *Category 2 (emotional) symptoms*,

c *Category 3 (cognitive) symptoms*,

d *Category 4 (insomnia)*.

*For all conditions, except for having no other pre-existing condition, all odds were greater for boys with the pre-existing condition compared to boys without that pre-existing condition, except for having no symptoms. For boys with no other pre-existing condition, all odds were less than boys with any pre-existing condition, except for having no symptoms*.

* *signifies statistical significance at the 0.05 level*,

** *signifies significance at the 0.01 level (Fisher's Exact Test)*.

**Table 2:** Percentages of girls with ADHD endorsing individual symptoms on the Post-Concussion Scale, stratified by pre-existing health condition and concussion history.

								**Concussion history**			
	**All girls** ** with** ** ADHD**	**No other** ** pre-existing** ** condition**	**Learning** ** disability**	**Academic** ** problems**	**Migraine**	**Psych.** ** Tx Hx**	**Substance** ** Tx Hx**	**None**	**1**	**2**	**3+**
	***n* = 885**	***n* = 299**	***n* = 190**	***n* = 185**	***n* = 91**	***n* = 294**	***n* = 14**	***n* = 710**	***n* = 106**	***n* = 27**	***n* = 29**
Headache^a^	**30.3**	21.7^**^	37.9^*^	**34.6**	56.0^**^	39.8^**^	**42.9**	**27.9**	**36.8**	51.9^*^	48.3^*^
Vomiting	4.4	2.7	4.2	4.9	7.7	6.1	7.1	4.4	3.8	3.7	6.9
Nausea^a^	10.7	5.7^**^	13.7	18.9^**^	18.7^*^	15.6^**^	14.3	9.3	17.0^*^	22.2^*^	17.2
Balance problems^a^	14.5	9.0^**^	18.4	16.8	26.4^**^	21.1^**^	42.9^**^	13.1	17.9	**22.2**	31.0^*^
Dizziness^a^	17.7	12.7^**^	24.2^*^	19.5	27.5^*^	24.8^**^	**28.6**	16.3	**23.6**	**29.6**	**27.6**
Trouble falling asleep^d^	**39.7**	30.4^**^	**44.7**	**42.2**	**50.5**	51.7^**^	**50.0**	**38.0**	**48.1**	**48.1**	**48.3**
Fatigue^a^	**33.4**	23.1^**^	**38.4**	**36.2**	**37.4**	44.6^**^	**35.7**	**31.5**	**35.8**	**40.7**	58.6^**^
Sleeping more than usual	11.2	8.0^*^	8.9	12.4	17.6	18.4^**^	14.3	10.7	15.1	11.1	10.3
Sleeping less than usual^d^	**35.8**	28.1^**^	**39.5**	**38.9**	**42.9**	43.9^**^	**42.9**	**34.8**	**38.7**	**44.4**	**41.4**
Sensitivity to light^a^	**20.8**	13.7^**^	27.4^*^	**23.8**	34.1^**^	27.6^**^	**21.4**	19.6	**21.7**	37.0^*^	**27.6**
Drowsiness	**26.2**	15.1^**^	34.2^**^	**31.4**	36.3^*^	36.1^**^	**42.9**	**24.5**	**30.2**	**40.7**	**37.9**
Sensitivity to noise^a^	16.4	10.0^**^	**20.5**	17.3	**23.1**	25.2^**^	7.1	16.3	12.3	**22.2**	**27.6**
Irritability^b^	**30.8**	18.1^**^	**35.8**	38.4^*^	**37.4**	49.0^**^	64.3^*^	**29.2**	**31.1**	**40.7**	65.5^**^
Nervousness^b^	**38.8**	28.8^**^	46.3^*^	45.9^*^	56.0^**^	55.8^**^	**50.0**	**40.0**	28.3^*^	**37.0**	**55.2**
Sadness^b^	**32.9**	16.7^**^	40.5^*^	42.2^**^	48.4^**^	53.1^**^	**57.1**	**31.3**	**35.8**	**40.7**	**48.3**
Feeling more emotional^b^	**35.7**	23.4^**^	47.4^**^	42.2^*^	49.5^**^	52.7^**^	**50.0**	**34.6**	**37.7**	**37.0**	**51.7**
Numbness or tingling	6.8	4.7	9.5	8.1	9.9	10.2^*^	28.6^*^	6.5	3.8	11.1	20.7^*^
Feeling mentally “Foggy”^c^	15.7	10.4^**^	15.8	16.2	**23.1**	25.2^**^	14.3	15.9	11.3	**22.2**	**20.7**
Feeling slowed down	17.3	9.7^**^	23.7^*^	22.7^*^	27.5^*^	26.2^**^	**21.4**	17.2	15.1	**25.9**	**24.1**
Difficulty concentrating^c^	**51.8**	45.8^**^	**56.8**	**54.6**	62.6^*^	61.9^**^	**78.6**	**51.0**	**49.1**	**66.7**	**69.0**
Difficulty remembering^c^	**22.5**	15.4^**^	33.7^**^	34.1^**^	**28.6**	31.0^**^	**35.7**	**22.3**	**20.8**	**29.6**	**34.5**
Visual problems	15.8	8.0^**^	21.6^*^	23.2^**^	28.6^**^	25.2^**^	50.0^**^	14.6	15.1	**22.2**	41.4^**^
No symptoms	18.9	27.1^**^	15.3	14.6	12.1	10.9^**^	0.0	**20.7**	14.2	7.4	0.0^**^
**Post-concussion scale**											
Mean	13.3	7.6	16.4	16.6	20.7	21.0	22.2	12.6	14.2	17.0	24.3
Median	7.0	4.0	12.0	11.0	19.0	18.0	21.0	7.0	8.0	10.0	21.0
SD	15.4	10.6	15.4	16.5	18.5	18.4	15.1	14.9	16.1	16.5	19.7
25th percentile	2.0	0.0	3.0	4.0	6.0	4.8	9.0	1.0	2.8	4.0	10.5
75th percentile	20.0	10.0	27.0	25.0	31.0	32.0	34.0	19.0	20.3	25.0	34.0
90th percentile	35.0	21.0	37.9	39.2	46.0	47.0	47.0	33.0	35.9	44.4	49.0

*All values are percentages endorsing the symptom as mild or greater. Symptoms endorsed by 20% or more of each group are bolded*.

*ADHD, Attention Deficit Hyperactivity Disorder; Tx, Treatment; Hx, History*.

*Symptoms counted toward each criterion for ICD-10 Post-concussional Syndrome*:

a *Category 1 (physical) symptoms*,

b *Category 2 (emotional) symptoms*,

c *Category 3 (cognitive) symptoms*,

d *Category 4 (insomnia)*.

*For all conditions, except for having no other pre-existing condition, all odds were greater for girls with the pre-existing condition compared to girls without that pre-existing condition, except for having no symptoms. For girls with no other pre-existing condition, all odds were less than girls with any pre-existing condition, except for having no symptoms*.

* *signifies statistical significance at the 0.05 level*,

** *signifies significance at the 0.01 level (Fisher's Exact Test)*.

### Statistical Analyses

We dichotomized the Post-Concussion Symptom Scale based on the ICD-10 symptom criteria for PCS. Namely, to meet ICD-10 PCS criteria, participants must endorse at least one symptom in at least three of the following categories: cognitive, somatic, emotional, and insomnia. Assignment of the specific symptoms to the respective category is denoted with superscripts in Tables 1, 2. The purpose of this study was to examine factors related to PCS-like symptom reporting in the absence of recent head trauma, so we did not apply the ICD-10 PCS criterion pertaining to head trauma history. Because the ICD-10 does not specify a threshold for symptom endorsement, we created more and less stringent versions. For “PCS-mild,” a rating of “mild” severity (i.e., item rating of one or greater on the Likert scale) was considered as symptom endorsement. Thus, a severity rating of one of greater for at least one symptom in at least three of the four categories would qualify a participant as meeting criteria for PCS-mild. Item ratings of at least “moderate” severity (i.e., three or greater) were considered as symptom endorsement toward a “PCS-moderate” classification. In other words, a severity rating of three or greater for at least one symptom in at least three categories would qualify a participant as meeting criteria for PCS-moderate.

Logistic regression was used to determine which covariates were associated with symptom reporting, using PCS-mild as the dichotomous outcome variable to maximize the ratio of participants with each outcome category to the number of covariates. Separate regressions were conducted for boys and girls. The following co-morbid conditions were included as covariates: learning disability (LD), prior special education services or having repeated a grade (Academic Problems), number of prior concussions (Prior Concussions), prior treatment for migraines, prior treatment for substance abuse, and prior treatment for a psychiatric condition. All covariates were entered in a single step for each model. Age was not included as a covariate because symptom reporting varies little across this narrow age range of adolescents (20).

## Results

The percentages of student athletes with ADHD who endorsed individual symptoms, stratified by gender, pre-existing health conditions, and concussion history, are presented in Tables 1, 2. Most adolescents with ADHD (69.3% of boys and 81.1% of girls) reported one or more symptoms. The most commonly reported symptoms in boys with ADHD were difficulty concentrating (38.4%), fatigue (28%), sleeping less than usual (25.7%), trouble falling asleep (22.5%), nervousness (19.1%), drowsiness (18.6%), headaches (18.3%), and irritability (17.9%). The most commonly reported symptoms in girls with ADHD were difficulty concentrating (51.8%), trouble falling asleep (39.7%), nervousness (38.8%), sleeping less than usual (35.8%), feeling more emotional (35.7%), fatigue (33.4%), sadness (32.9%), irritability (30.8%), and headaches (30.3%). Adolescents with ADHD who had additional pre-existing conditions, such as prior treatment for a psychiatric condition, prior treatment for migraine, or a history of treatment for substance abuse, endorsed considerably more physical, emotional, and cognitive symptoms compared to adolescents with ADHD only.

Fisher's exact tests were conducted to compare rates of individual symptom endorsement between adolescents with ADHD and another pre-existing condition compared to those without the pre-existing condition (see Tables 1, 2). In both boys and girls with ADHD, having a history of treatment for a psychiatric condition was associated with higher odds of endorsing every individual symptom, except for “vomiting.” For student athletes with ADHD who reported no other pre-existing condition (i.e., they have ADHD only), the odds of endorsing every individual symptom were lower than student athletes who reported having any additional pre-existing condition, except for “vomiting” and “numbness and tingling” in girls.

**Figure 1:**
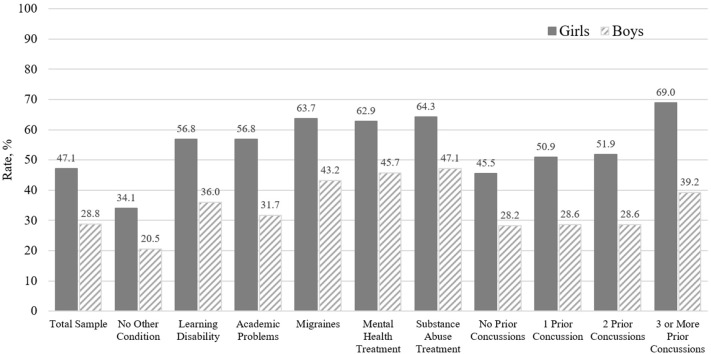
Rates of international classification of diseases, 10th revision (ICD-10), post-concussional syndrome classification in adolescent athletes with ADHD and no recent concussion (mild or greater symptoms in each domain).

A substantial proportion of boys (28.8%) and girls (47.1%) with ADHD endorsed a cluster of symptoms similar to ICD-10 symptom criteria for PCS-mild (see Figure 1), and 8.6% of boys and 22.1% of girls with ADHD met ICD-10 symptom criteria for PCS-moderate. Moreover, a sizable proportion of subgroups of boys with ADHD who also had a history of treatment for substance abuse (47.1%), a psychiatric condition (45.7%), or migraines (43.2%); three or more prior concussions (39.2%); or a co-occurring learning disability (36.0%) endorsed a cluster of symptoms consistent with PCS-mild. Similarly, high rates of PCS-mild were present in girls with ADHD who also have a history of treatment for migraines (63.7%), a psychiatric condition (62.9%), or substance abuse (64.3%); three or more prior concussions (69.0%); or a co-occurring learning disability (56.8%). Of note, among adolescents with ADHD who did not have any of the pre-existing conditions (i.e., they have ADHD only; *n* = 941), the frequency of PCS-mild was 20.5% for boys and 34.1% for girls; the frequency of PCS-moderate was only 4.9% for boys and 8.4% for girls.

Six predictor variables (listed in Table 3) were entered in a logistic regression for boys and five predictors (listed in Table 4) were entered for girls, with PCS-mild classification as the dichotomous outcome (prior treatment for substance abuse was omitted from the model for girls because there were only five non-events in girls with a history of substance abuse treatment; i.e., only five girls did not meet the PCS-mild criteria). The logistic regression was conducted with 1,852 boys [excluding 294 (13.7%) with at least one missing data point] and 762 girls [excluding 123 (13.9%) with at least one missing data point]. We examined the sources of missing data and compared participants with complete data (and who therefore were included in the logistic regression analysis) vs. those with incomplete data. Most excluded boys and girls were missing multiple data points (median = four missing items), typically the treatment history variables (e.g., history of treatment for a psychiatric condition or substance use). Boys excluded from the analysis were more likely to meet PCS-mild criteria (36.1 vs. 27.6%; $\chi_{\left(1\right)}^{2}$ = 8.75, *p* = 0.003) and were more likely to report having never sustained a prior concussion [88.7 vs. 74.1%; $\chi_{\left(1\right)}^{2}$ = 26.24, *p* < 0.001]. Girls who were excluded had a similar rate of PCS-mild classification (41.5 vs. 48%) and a similar rate of having never sustained a prior concussion (80.8 vs. 85.5%) compared to girls included in the model.

**Table 3:** Logistic regression predicting mild ICD-10 post-concussional syndrome for boys with ADHD.

	**Unadjusted**	**Adjusted**	**Bootstrapped**	
	**Odds ratio (CI)**	**Odds ratio (CI)**	**Odds ratio (CI)**	**% with mild ICD-10 post-concussional syndrome**
LD	1.63 (1.28–2.09)^**^	1.41 (1.07–1.86)^*^	1.41 (1.06–1.89)^*^	LD absent: 27.1%LD present: 36.0%
Academic problems	1.30 (1.05–1.62)^**^	1.03 (0.81–1.32)	1.03 (0.81–1.33)	Academic problems absent: 27.6%Academic problems present: 31.7%
Prior concussions	1.10 (0.97–1.26)	0.99 (0.87–1.14)	0.99 (0.87–1.13)	0: 28.2% 1: 28.6% 2: 28.6% 3+: 39.2%
Tx for migraines	2.08 (1.49–2.90)^**^	1.91 (1.35–2.69)^**^	1.91 (1.33–2.68)^**^	Migraine tx hx absent: 26.4% Migraine tx hx: present: 43.2%
Tx for substance abuse	2.29 (1.37–3.83)^**^	1.59 (0.92–2.71)	1.59 (0.88–2.80)	Substance abuse tx hx absent: 27.3% Substance abuse tx hx: present: 47.1%
Tx for psych. condition	2.70 (2.12–3.42)^**^	2.44 (1.91–3.12)^**^	2.44 (1.93–3.17)^**^	Psych. condition tx hx absent: 23.6% Psych. condition tx hx: present: 45.7%

*CI, 95% confidence interval; LD, Learning disability; ADHD, Attention Deficit Hyperactivity Disorder; Tx, Treatment*.

*Symptom ratings were derived from the Post-Concussion Symptom Scale, a dimensional scale that includes 22 items that are rated from zero to six, with 1–2 being mild, 3–4 being moderate, and 5–6 being severe. No athlete reported sustaining a concussion in the past 6 months. Therefore, these percentages should be considered the rates of presumed false positive diagnoses of mild post-concussional syndrome (however, it is possible that some of the athletes with a history of concussion could be conceptualized as true positive diagnoses—this is unknowable, however, because these are preseason survey data). To meet diagnostic criteria, the student must endorse one or more symptoms in 3 out of 4 symptom domains: Physical Symptoms (headache, dizziness, balance problem, noise sensitive, light sensitive, and/or fatigue), Emotional Symptoms (irritability, sadness, nervousness, and/or feeling more emotional), Cognitive Symptoms (poor concentration, poor memory, and/or feeling mentally foggy), and Insomnia (trouble falling asleep and/or sleeping less than usual). The symptom domain must have been endorsed as simply “present” (i.e., mild or greater symptoms) or as moderate or severe*.

* *signifies statistical significance at the 0.05 level*,

** *signifies significance at the 0.01 level*.

**Table 4:** Logistic regression predicting mild ICD-10 post-concussional syndrome for girls with ADHD.

	**Unadjusted**	**Adjusted**	**Bootstrapped**	
	**Odds ratio (CI)**	**Odds ratio (CI)**	**Odds ratio (CI)**	**% with mild ICD-10 post-concussional syndrome**
LD	1.69 (1.19–2.40)^**^	1.31 (0.89–1.94)	1.31 (0.84–1.99)	LD absent: 44.5% LD present: 56.8%
Academic problems	1.64 (1.15–2.32)^**^	1.31 (0.89–1.93)	1.31 (0.87–1.95)	Academic problems absent: 44.6% Academic problems present: 56.8%
Prior concussions	1.31 (1.06–1.61)^*^	1.15 (0.92–1.43)	1.15 (0.92–1.47)	0: 45.5% 1: 50.9% 2: 51.9% 3+: 69.0%
Tx for migraines	1.84 (1.14–2.98)^*^	1.35 (0.81–2.25)	1.35 (0.77–2.36)	Migraine tx hx absent: 46.6% Migraine tx hx: present: 63.7%
Tx for substance abuse	(n too small to run)	(not included in model)	(not included in model)	Substance abuse tx hx absent: 47.8% Substance abuse tx hx: present: 64.3%
Tx for psych. condition	2.70 (1.98–3.69)^**^	2.40 (1.74–3.31)^**^	2.40 (1.77–3.36)^**^	Psych. condition tx hx absent: 52.4% Psych. condition tx hx: present: 62.9%

*CI, 95% confidence interval; LD, Learning disability; ADHD, Attention Deficit Hyperactivity Disorder; Tx, Treatment*.

*Symptom ratings were derived from the Post-Concussion Symptom Scale, a dimensional scale that includes 22 items that are rated from zero to six, with 1-2 being mild, 3-4 being moderate, and 5–6 being severe. No athlete reported sustaining a concussion in the past 6 months. Therefore, these percentages should be considered the rates of presumed false positive diagnoses of mild post-concussional syndrome (however, it is possible that some of the athletes with a history of concussion could be conceptualized as true positive diagnoses—this is unknowable, however, because these are preseason survey data). To meet diagnostic criteria, the student must endorse one or more symptoms in 3 out of 4 symptom domains: Physical Symptoms (headache, dizziness, balance problem, noise sensitive, light sensitive, and/or fatigue), Emotional Symptoms (irritability, sadness, nervousness, and/or feeling more emotional), Cognitive Symptoms (poor concentration, poor memory, and/or feeling mentally foggy), and Insomnia (trouble falling asleep and/or sleeping less than usual). The symptom domain must have been endorsed as simply “present” (i.e., mild or greater symptoms) or as moderate or severe*.

* *signifies statistical significance at the 0.05 level*,

** *signifies significance at the 0.01 level*.

For boys, the logistic regression model was statistically significant (*p* < 0.001) and well-calibrated (Hosmer and Lemeshow, *p* = 0.68). Measures of discrimination suggested modest prediction accuracy (Nagelkerke *R*^2^ = 0.068; area under the receiver operating curve (AUC) = 0.62, *p* < 0.001, i.e., better than chance but poor for classifying individual cases). For girls, the logistic regression model was also statistically significant (*p* < 0.001) and well-calibrated (Hosmer and Lemeshow *p* = 0.55). Measures of discrimination again suggested modest prediction accuracy (Nagelkerke *R*^2^ = 0.085; AUC = 0.64, *p* < 0.001).

As seen in Table 3 for boys and Table 4 for girls, all covariates except for prior concussions in boys had a significant relationship with PCS classification when considered in univariate models. In the adjusted model for boys, the significant predictors (in descending order based on odds ratios) were: past treatment for a psychiatric condition, treatment for migraines, and co-occurring LD. In the adjusted model for girls, treatment for a psychiatric condition was the only significant predictor of meeting criteria for PCS. Bootstrapping was performed to assess risk of model over-fitting for the adjusted models. All results from the adjusted bootstrapped model were consistent with results from the adjusted model.

## Discussion

This is the largest study to date examining concussion-like symptom reporting among uninjured adolescent athletes with ADHD. Using a dataset of over 48,000 adolescent student athletes, we studied symptom reporting among more than 3,000 adolescents with self-reported ADHD. Because only a small percentage of student athletes have ADHD, massive numbers of students must be monitored to study adequate sample sizes and to allow for stratification of youth with ADHD by multiple co-occurring health conditions, as we were able to do here. Consistent with our hypotheses, we found that most student athletes with ADHD endorse concussion-like symptoms during baseline, preseason assessment, in the absence of recent injury. Girls and adolescents with ADHD who also reported additional, co-occurring problems and conditions (e.g., prior treatment for a psychiatric condition or migraines) reported greater symptoms. Moreover, a substantial proportion of adolescents with ADHD endorsed a combination of physical, emotional, cognitive, and sleep-related symptoms that resemble an ICD-10 diagnosis of PCS (see Figure 1). Boys with co-occurring learning disorder as well as those with prior treatment for migraines and prior treatment for a psychiatric condition were more likely to meet criteria for PCS. For girls, only prior treatment for a psychiatric condition was independently associated with increased odds of meeting criteria for PCS.

Prior treatment for a psychiatric condition had the largest effect size with regard to symptom reporting among both boys and girls with ADHD. This was also the case when considering the broader population of adolescents, not just those with ADHD (14). The pattern of symptoms endorsed by adolescents with ADHD did not necessarily align with the particular pre-existing condition in question. It was not the case, for example, that participants with ADHD and a history of treatment for a psychiatric condition selectively reported more emotional health-related symptoms, such as sadness and nervousness. Participants with ADHD and co-occurring health conditions, and even participants with ADHD only (i.e., without additional health conditions), endorsed a wide variety of symptoms across emotional, cognitive, sleep, and physical/somatic domains. In previous studies (14, 21) a positive association has been noted between prior concussions and increased preseason, baseline symptom reporting. Our study replicated this finding but revealed that, among adolescents with ADHD, prior concussions are not independently associated with symptom reporting.

The results of this study have implications regarding the clinical care for youth with ADHD. In a recent survey, the vast majority of pediatric primary care providers reported they typically allow a patient to return to physical activity or sports when they are completely asymptomatic (87% of respondents) (11). Our results caution against such an approach because many youth with ADHD are “symptomatic” in the absence of head injury. These symptoms can be associated with stress (22), feeling depressed (23), and insufficient sleep (24). Moreover, some symptoms might be, to a certain degree, long standing and dispositional for some student athletes. Waiting for complete symptom resolution may delay an adolescent's return to activities, which might further complicate their recovery. In regard to using the Post-Concussion Symptom Scale within ImPACT® in clinical practice, this study provides normative data for symptom reporting among youth with ADHD stratified by sex and other pre-existing health conditions to help clinicians interpret symptoms in student athletes with ADHD who sustain a concussion.

The percentile ranks presented in Tables 3, 4 can be considered thresholds for clinical interpretation. We provide normative lookup tables for girls (Table 5) and boys (Table 6) with ADHD based on the percentile ranks. For these tables, scores that fell at or below the 75th percentile were considered “Broadly normal;” scores that fell between the 75th and 90th percentiles were considered “above normal;” and scores that fell above the 90th percentile were considered “unusually high.” In clinical practice, if a girl with ADHD and no other comorbidities completed the PCS and obtained a score of 22 (see Table 5), that could be considered a significantly elevated result (i.e., above the 90th percentile and in the Unusually High range). In contrast, if a girl with ADHD also had prior treatment for migraines, prior treatment for a psychiatric condition, or a learning disability, a PCS score of 22 falls within the broadly normal range (i.e., below the 75th percentile). Similarly, for a boy with ADHD and no other conditions or prior injuries (see Table 6), a PCS score of 15 is “unusually high,” whereas for a boy with ADHD who also has a prior history of migraine or treatment for a psychiatric condition this score would be in the broadly normal range.

**Table 5:** Normative ranges for post-concussion scale total symptom severity score for girls with ADHD, stratified by pre-existing health condition and concussion history.

**Reference group**	**Classification**		
	**Broadly normal**	**Above normal**	**Unusually high**
All girls with ADHD	0–20	21–35	36+
No other pre-existing condition	0–10	11–21	22+
Learning disability	0–27	28–37	38+
Academic problems	0–25	26–39	40+
Migraine	0–31	32–46	47+
Psych Tx Hx	0–32	33–47	48+
Substance Tx Hx	0–34	35–47	48+
No prior concussion	0–19	20–33	34+
1 prior concussion	0–20	21–35	36+
2 prior concussions	0–25	26–44	45+
3 or more prior concussions	0–34	35–49	50+

*The “Broadly normal” scores fell at or below the 75th percentile. The “above normal” scores fell between the 75th and 90th percentiles. The “unusually high” scores fell above the 90th percentile*.

**Table 6:** Normative ranges for post-concussion scale total symptom severity score for boys with ADHD, stratified by pre-existing health condition and concussion history.

**Reference group**	**Classification**		
	**Broadly normal**	**Above normal**	**Unusually high**
All boys with ADHD	0–10	11–20	21+
No other pre-existing condition	0–6	7–14	15+
Learning disability	0–14	15–27	28+
Academic problems	0–12	13–26	27+
Migraine	0–17	18–30	31+
Psych Tx Hx	0–19	20–30	31+
Substance Tx Hx	0–17	18–28	29+
No prior concussion	0–9	10–19	20+
1 prior concussion	0–10	11–19	20+
2 prior concussions	0–13	14–26	27+
3 or more prior concussions	0–16	17–33	34+

*The “Broadly normal” scores fell at or below the 75th percentile. The “above normal” scores fell between the 75th and 90th percentiles. The “unusually high” scores fell above the 90th percentile*.

This study has several limitations. First, ADHD status and all other health history variables were determined via adolescent self-report. We were not able to verify this information through parent corroboration or medical chart review. With that said, self-reported health history information may be the only source available for school-based preseason testing and rapid screening assessments in certain clinical situations. Moreover, a prior study illustrated that adolescents who undergo baseline testing twice, separated by an average of 2 years, are highly consistent in reporting a personal history of ADHD (25). Second, there are multiple challenges regarding the identification and classification of pediatric PCS (26). We applied the ICD-10 criteria but several sets of diagnostic criteria for pediatric PCS have been proposed and more research is needed to validate and refine these criteria in children (16). Third, although our regression models identified factors associated with baseline symptom reporting, they are not sufficient to predict the presence or absence of meeting PCS criteria in an individual athlete based on their sex and pre-existing health conditions. Many other factors are associated with symptom reporting in adolescents and young adults, such as life stress (22) and insufficient sleep (24).

In conclusion, many student athletes with ADHD and no recent concussion, even those without any additional pre-existing conditions, report a cluster of symptoms on baseline assessment that resemble a mild version of the ICD-10 post-concussional syndrome. Thus, ADHD appears to “mimic” the post-concussional syndrome in many adolescents. This study adds to a growing literature highlighting that concussion-like symptoms are non-specific (27–29), and are frequently reported by uninjured adolescents with ADHD (13, 14, 30–32). Moreover, no prior study has stratified symptom reporting among youth with ADHD based on several additional health conditions. Having a co-occurring learning disorder as well as prior treatment for a psychiatric condition or migraine were associated with greater symptom reporting compared to youth with ADHD and no other health conditions. The results of this study emphasize that “asymptomatic” status after concussion can be challenging to define (33), and may not always be a reasonable goal in all young athletes, especially in youth with ADHD. Health care providers evaluating youth with ADHD who have slow recovery from a concussion are encouraged to carefully and thoughtfully discuss current symptoms in the context of their life and try to disentangle the extent to which they might be related to lingering physiological problems due to concussion vs. other factors.

## Data Availability Statement

The data analyzed in this study was obtained from the Maine Concussion Management Initiative. Requests to access these datasets should be directed to Dr. Paul Berkner, paul.berkner@colby.edu.

## Ethics Statement

The studies involving human participants were reviewed and approved by Colby College Institutional Review Board (IRB). Written informed consent from the participants' legal guardian/next of kin was not required to participate in this study in accordance with the national legislation and the institutional requirements.

## Author Contributions

NC, NS, and GI contributed conception and design of the study. BM organized the database. NC and RS performed the statistical analysis. NC wrote the first draft of the manuscript. RS and GI wrote sections of the manuscript. All authors contributed to manuscript revision, read and approved the submitted version.

### Conflict of Interest

GI serves as a scientific advisor for BioDirection, Inc., Sway Operations, LLC, and Highmark, Inc. He has received research funding from several test publishing companies, including ImPACT Applications, Inc., CNS Vital Signs, and Psychological Assessment Resources (PAR, Inc.). He has received research funding as a principal investigator from the National Football League, and salary support as a collaborator from the Harvard Integrated Program to Protect and Improve the Health of National Football League Players Association Members. RZ has received salary support from the Harvard Integrated Program to Protect and Improve the Health of National Football League Players Association Members. RZ serves on the Scientific Advisory Board of Myomo, Oxeia Pharma, and ElMInda. The remaining authors declare that the research was conducted in the absence of any commercial or financial relationships that could be construed as a potential conflict of interest.
